# Ramifications of untreated hypothyroidism: case report of cognitive impairment and acute psychosis in an elderly female

**DOI:** 10.1186/s12991-020-00300-8

**Published:** 2020-09-05

**Authors:** Janette C. Leal, Allison H. Beito

**Affiliations:** Department of Psychiatry and Psychology, Mayo Clinic, 200 First St SW, Rochester, MN 55905 USA

## Abstract

Hypothyroidism is a common condition in the general population. While myxedema is a known complication, we present a case highlighting a late-onset presentation of psychosis with confounding cognitive impairment in a patient who believed she no longer needed thyroid replacement medication because of her advanced age.

## Introduction

Hypothyroidism is a common condition in the older population, estimated to afflict 5–20% of elderly women [1]. With an aging population, cases are expected to become more widespread in the near future. While psychiatric presentations of hypothyroidism were initially described in the late 1880s, and the term “myxedema madness” was added to the literature in 1949 [2, 3], ensuing cases over the years have highlighted the true diversity of potential psychotic presentations. Elderly patients in particular may demonstrate affective disturbance, cognitive dysfunction, and a wide range of psychotic symptoms, without a predictable progression of medical or psychiatric symptoms [2, 3]. Psychiatric presentations of hypothyroidism require a vigilant approach to investigation for thyroid disturbance, as left untreated, hypothyroidism may lead to excess morbidity.

Here, we present one case of hypothyroidism-related psychosis and cognitive impairment treated in our hospital, with the purpose of further illustrating the range of potential presentations of which providers should be aware.

## Case presentation

A 79-year-old, married, Caucasian female was brought to medical attention by her husband. She had suffered 2 weeks of escalating delusions of pregnancy, hallucinations with observed response to internal stimuli at home, and agitation with increasing suspiciousness toward family members. Her previous medical history was pertinent for treated hypothyroidism of 50 years duration, hyperlipidemia, diverticulosis, Vitamin D deficiency, and mild osteoarthritis. She had no family or personal history of formally diagnosed psychiatric disorders. She was high school graduate, retired housecleaner and teacher’s aide, who lived independently in a house with her husband. She had no lifetime history of any substance use. Interestingly, she had been hospitalized 1 year prior to this presentation, for uncontrolled hypothyroidism and paranoid thoughts associated with medication non-adherence. At that time, thyroid replacement had been re-initiated with improvement in paranoia.

Prior to this admission, the patient insisted that she had been impregnated by an intruder into her home, and possessed delusional thoughts that her abdomen was filled with embryos, necessitating caesarian section. She was willing to seek medical attention only for the purpose of scheduling a caesarian section. Her husband observed her speaking to family members who were not present, and she referred to ideas of reference, that she was hearing the voices of individuals from nearby towns, which were broadcasted over her television, and also surveilling her. She possessed a delusional thought that her sister was making plans to bomb her home. She endorsed stopping her home levothyroxine dose (100 mcg daily) 2 weeks prior, and expressed conviction that she no longer needed the medication, due to her advanced age, after hearing that a friend had discontinued their own levothyroxine.

Upon presentation, the patient complained only of mild abdominal discomfort “fullness”, but otherwise had no other physical nor specific psychiatric complaints. She denied concerns with mood. She believed she was in the Emergency Department for a caesarian section. She was unable to coherently provide a history of present illness, insisting that she was presenting for surgery, and making other unrelated delusional comments “I took a fireball to the head.” She possessed no vital sign abnormalities, with heart rate of 63 and blood pressure of 140/69. She had no obvious physical exam abnormalities, including dry skin, thinning hair, edema, hoarseness, goiter, or macroglossia. Cognitive screening revealed deficiencies in calculation and delayed recall with Mental Status Exam [4] score of 25/38 (with construction not performed), despite full orientation and level of alertness. Lab evaluations revealed no abnormalities in blood count or electrolytes, however TSH was elevated to 403 mIU/L, free T4 was undetectable, and T3 was diminished at 1.7 ng/dL. TPO antibodies were not collected. She also demonstrated elevated total serum cholesterol (224 mg/dL) and triglycerides (303 mg/dL). Serum vitamin D levels were diminished at 20 ng/mL. Workup was negative for infections including a normal urinalysis, CBC and BMP.

The patient was treated with initial dosing of IV levothyroxine and was then transitioned back to PO levothyroxine at 100 mcg daily. Vitamin D replacement was initiated. She demanded to leave the hospital, and after determination that she did not possess decision-making capacity secondary to psychosis, she was placed on a psychiatric hold and the treatment team petitioned her home county for mental health commitment. She was started on therapy with olanzapine 10 mg daily, for ongoing paranoia and delusional thoughts. Her cognitive screening score had improved marginally to 30/38 by her admission to inpatient psychiatry, though still with difficulty with delayed recall and calculation.

Throughout her month-long psychiatric stay, and with ongoing treatment with levothyroxine and olanzapine, our patient’s psychotic symptoms slowly improved. Her delusion of pregnancy resolved, and she exhibited no further hallucinations or ideas of reference. Her TSH improved to a level of 64 mIU/L, and free T4 returned to normal range.

After discharge, the patient returned home, and discontinued her medications (levothyroxine and olanzapine) as she continued to believe that she is old and no longer needed it. She was re-hospitalized a month later after presenting to the emergency department with abdominal pain requesting to have “babies flushed out of her”. Per chart review, she has been medication complaint since, living at home with her husband and following up periodically with her PCP.

## Discussion

Hypothyroidism, an extremely common medical condition, is particularly widespread among female and elderly patients, afflicting between 5 and 20% of elderly females [1]. While medical sequelae, including dyslipidemia, constipation, cold intolerance, fatigue, thinning hair, hoarseness, joint and muscle pain, and myxedema are commonly appreciated, the physical signs and symptoms in elderly patients may be mild, absent or atypical [1]. Psychiatric manifestations of hypothyroidism were initially recognized in the late 1880s, and include mood disturbance, cognitive impairment, and psychotic symptoms. While the mechanism is not fully understood, it is appreciated that a large percentage of thyroid hormone receptors reside in the brain (in the amygdala and hippocampus), and decreased conversion from T4 to T3 (active thyroid hormone) in elderly patients could be one mechanism predisposing this cohort to neuropsychiatric symptoms [1, 2].

Asher first coined the term ‘myxedema madness’ in his 1949 case series [2, 3], describing 14 different presentations of psychosis (most in older females) secondary to hypothyroidism. Even 70 years ago, he emphasized that this was a common phenomenon, which was often unrecognized by medical practitioners. Later estimates indicate that up to 15% of patients with hypothyroidism will present with psychiatric, rather than medical, symptoms [5]. A 1999 investigation of admissions of elderly patients to a geriatric psychiatry unit, found that over 1/3 of them had unrecognized medical conditions, with the top three including hypothyroidism [6]. Later case reports have described symptoms ranging from paranoia, delusions, and hallucinations, to catatonia, Capgras syndrome, and even mania in elderly patients with untreated hypothyroidism [7–9]. While some patients experience concomitant medical signs and symptoms, in many patients, the psychiatric or behavioral disturbance is the sole manifestation of an underlying endocrine disorder. We were fortunate in this case, that the patient had a clear history of hypothyroidism preceding the onset of psychotic symptoms, and a clear collateral history provided by family indicating she had become non-adherent to thyroid replacement. This prompted investigation of thyroid hormone levels immediately on presentation, and thyroid replacement was initiated without delay. In other circumstances, diagnosis can be more difficult. While the patient had a history of mild paranoid personality traits, she, like several other patients described in case studies, arrived to medical attention with a first episode of psychosis in older age, which should prompt an investigation of an organic etiology rather than attribution of symptoms to idiopathic psychiatric disturbance. The most challenging aspect of the case lied in the development of delusions specifically surrounding thyroid hormone replacement, in that the patient maintained conviction that she no longer needed to take her levothyroxine. This delusion persisted even after initiation of treatment, and left her vulnerable to non-adherence, relapse of psychotic symptoms, and medical morbidity. While her county commitment included provisions for Jarvis petition with mandatory administration of antipsychotic medications, no provision can mandate adherence to thyroid replacement therapy.

In hypothyroidism-induced psychiatric symptoms, thyroid replacement remains the gold-standard in treatment, however even with proper replacement, symptoms may take several months to abate [5]. For this reason, several providers in later case reports have described using short-term treatment with antipsychotics to ease distressing psychotic symptoms. The use of haloperidol, olanzapine, and risperidone has been described with success [5, 7–9]. In this case, we found concomitant treatment with olanzapine and levothyroxine to be efficacious in reduction of psychotic symptoms, although it was impossible to quantify how much benefit was attributed to the antipsychotic. As the patient relapsed in follow-up prior to normalization of her thyroid hormone levels, we are unable to recommend a specific duration of therapy with antipsychotics as they were not able to be successfully tapered in the outpatient setting.

Similar to uncertainty the mechanism of development of neuropsychiatric symptoms of hypothyroidism, there remains question surrounding the degree of reversibility of these symptoms when adequate thyroid replacement has been achieved. Asher observed that a few of his original case subjects had persistent psychotic symptoms even after treatment [3]. It is theorized that metabolic impairment secondary to hypothyroidism may have the potential to cause irreversible CNS damage related to blood flow abnormalities and/or glucose metabolism [7]. We unfortunately did not observe a full restoration of our patient to previous level of cognitive function, as her delusional thoughts surrounding thyroid hormone replacement persisted. We were unable to obtain follow-up cognitive screening after hospitalization, and it was therefore uncertain if her mild cognitive deficits in delayed recall and calculation were a product of psychosis, cognitive impairment secondary to hypothyroidism, or an underlying cognitive impairment that had not been previously elucidated.

## Conclusion

Hypothyroidism remains a common and important organic cause of neuropsychiatric symptoms, particularly in elderly female patients. All elderly patients presenting with cognitive decline and new-onset psychiatric or behavioral disturbance warrant screening of thyroid status as a potentially contributing factor. While thyroid replacement remains the standard treatment, addition of antipsychotic medications may be helpful to ease particularly distressing and long-lived symptoms in elderly patients. The optimal duration of antipsychotic therapy is unknown. This, in addition to several other case studies, illustrates the diversity of possible presentations of ‘myxedema madness’, heightening the need for vigilance in screening and diagnosis in order to prevent treatment delay and patient morbidity.
